# Biocontrol and Action Mechanism of *Bacillus subtilis* Lipopeptides’ Fengycins Against *Alternaria solani* in Potato as Assessed by a Transcriptome Analysis

**DOI:** 10.3389/fmicb.2022.861113

**Published:** 2022-05-11

**Authors:** Dai Zhang, Ran Qiang, Zhijun Zhou, Yang Pan, Shuiqing Yu, Wei Yuan, Jianing Cheng, Jinhui Wang, Dongmei Zhao, Jiehua Zhu, Zhihui Yang

**Affiliations:** ^1^College of Plant Protection, Hebei Agricultural University, Baoding, China; ^2^Practice and Training Center, Hebei Agricultural University, Baoding, China; ^3^Hebei Pingquan Edible Fungi Industry Technology Research Institute, Chengde, China; ^4^Agricultural Business Training and Entrepreneurship Center, Hebei Agricultural University, Baoding, China

**Keywords:** *Alternaria solani*, *Bacillus subtilis*, fengycins, transcriptome, cell wall and membrane, conidia

## Abstract

*Alternaria solani* is an airborne fungus and the primary causal agent of potato early blight worldwide. No available fungicides that are both effective and environmentally friendly are usable to control this fungus. Therefore, biological control is a potential approach for its suppression. In this study, *Bacillus subtilis* strain ZD01’s fermentation broth strongly reduced *A. solani* pathogenicity under greenhouse conditions. The effects of strain ZD01’s secondary metabolites on *A. solani* were investigated. The exposure of *A. solani* hyphae to the supernatant resulted in swelling and swollen sacs, and the ZD01 supernatant reduced *A. solani* conidial germination significantly. Matrix-assisted laser desorption/ionization time of flight mass spectrometry and pure product tests revealed that fengycins were the main antifungal lipopeptide substances. To elucidate the molecular mechanism of the fengycins’ biological control, RNA sequencing analyses were performed. A transcriptome analysis revealed that 304 and 522 genes in *A. solani* were differentially expressed after 2-h and 6-h fengycin treatments, respectively. These genes were respectively mapped to 53 and 57 Kyoto Encyclopedia of Genes and Genomes (KEGG) pathways. In addition, the most enriched KEGG pathway analysis indicated that the inhibitory mechanisms of fengycins against *A. solani* regulated the expression of genes related to cell wall, cell membrane, transport, energy process, protein synthesis and genetic information. In particular, cell wall and cell membrane metabolism were the main processes affected by fengycin stress. Scanning and transmission electron microscope results revealed hyphal enlargement and a wide range of abnormalities in *A. solani* cells after exposure to fengycins. Furthermore, fengycins induced chitin synthesis in treated cells, and also caused the capture of cellular fluorescent green labeling and the release of adenosine triphosphate (ATP) from outer membranes of *A. solani* cells, which may enhance the fengycins ability to alter cell membrane permeability. Thus, this study increases the transcriptome data resources available and supplies a molecular framework for *B. subtilis* ZD01 inhibition of *A. solani* HWC-168 through various mechanisms, especially damaging *A. solani* cell walls and membranes. The transcriptomic insights may lead to an effective control strategy for potato early blight.

## Introduction

Early blight, caused by the fungus *Alternaria solani*, is an important and frequent disease of potatoes worldwide. Early blight disease on potato can cause up to 80% annual yield losses in some regions (Morgan et al., 2002; Pasche et al., 2007). Conventional chemical fungicides are highly effective against this pathogen and; therefore, are commonly used to control early blight disease on potato (Davidson et al., 2015). However, frequent applications of fungicides result in environmental concerns and fungicide resistance in *A. solani* (Wei et al., 2019). Due to this, there is an increasing need for alternative environmentally friendly effective methods to control potato early disease.

*Bacillus* species have been widely used in the biological control of plant pathogens owing to their strong inhibitory activities against various pathogenic fungi (Jiang et al., 2020). Consequently, they show great potential as applications in agricultural fields. For example, *B. amyloliquefaciens* and *B. subtilis* control *Phytophthora sojae* by inhibiting mycelial growth and zoospore germination (Liu et al., 2008). Lipopeptides (LPs) are the major antifungal compounds produced by *Bacillus* strains, and they modify cell membrane permeability, induce systemic resistance in plants, and are involved in biofilm formation. The LPs produced by *Bacillus* strains represent important tools in the development of new effective products against plant pathogens (Rodríguez-Chávez et al., 2019).

There are three families in LPs, including the surfactins, iturins and fengycins. Among them, fengycins exhibit strong anti-fungal activities against plant pathogens, specifically against filamentous fungi (Velho et al., 2011; Zhang and Sun, 2018). Fengycins from *Bacillus* strains play important roles in the inhibition of *Monilinia laxa*, *Monilinia fructicola*, *Rhizoctonia solani*, *Botryosphaeria dothidea*, *Fusarium moniliforme*, and *Fusarium graminearum* (Hu et al., 2007; Yánez-Mendizábal et al., 2012; Guo et al., 2014; Fan et al., 2017; Hanif et al., 2019). In addition, fengycins cause ultrastructural destruction of the fungal pathogen hyphae. Thus, fengycin-treated hyphae exhibit unconsolidated cytoplasm, and the cell walls are gapped and/or separated from the cell membrane (Deleu et al., 2008; González-Jaramillo et al., 2017; Zhang et al., 2018).

Transcriptome analyses use a rapid and high-throughput technology for the quantification and identification of gene expression. At present, transcriptomes have been popularly applied to reveal changes in an organism’s gene expression patterns in different environments (Nookaew et al., 2012; OuYang et al., 2016; Zhang et al., 2016, 2018). Moreover, they have also been used to explore the molecular mechanisms of fungal drug-resistance or fungi–host interactions (Liu et al., 2015; Barad et al., 2016; Wang et al., 2016). The fungal response mechanisms to antimicrobial chemicals, such as iturins, C_12_-OOWW-NH_2_, and other antimicrobial substances have been successfully investigated (Muthaiyan et al., 2008; Pietiäinen et al., 2009; Kim et al., 2016; Le et al., 2016; Sun et al., 2017; Wang et al., 2017; Zhao et al., 2017; Jiang et al., 2020; Li et al., 2020).

Although fengycins have shown promising antifungal activities, the antagonistic effects, and related mechanisms, of fengycins against *A. solani* remain mostly unknown. Limited studies have focused on the biocontrol effects of identified specific compounds of LPs secreted by *Bacillus* strains against *A. solani* in potato using transcriptomes. In the current study, we aimed to investigate the molecular antifungal mechanisms of fengycins against *A. solani* using transcriptomes and to identify the effects of fengycins on the cell walls and membranes of *A. solani*.

## Materials and Methods

### Strains and Culture Conditions

The dual-culture assay on PDA (Hu et al., 2014) was used to test bacterial antagonistic activities against *A. solani*. The plugs (5 mm in diameter) of plant the pathogenic fungus were placed onto the centers of PDA plates. The ZD01 isolates (5 μL, 1 × 10^8^ CFU/mL) were inoculated 2 cm away from plant fungus. Four isolates were spotted in each dish, and a blank control of sterile water was used. All the fungal pathogens used are listed in Supplementary Table 1. All the isolates were tested in triplicate, and their inhibition zones were measured after 7 days of dual culture at 25°C. The inhibition rate on mycelial growth was calculated using the following formula:


Inhibitionrateonmycelialgrowth(%)=(thediameterofcontrol-thediameteroftreatment)/thediameterofcontrol×100%.

### Pot Assay Under Greenhouse Conditions Using Strain ZD01 Fermentation Broth

The potato seeds were planted in vermiculite:nutritive soil (1:3, v/v) in 17.5-cm diameter plastic pots. Each group was replicated using eight pots. All the experiments were performed in a greenhouse cabinet at 25°C in the light for 14 h/day and at 18°C in the dark for 10 h/day. After potato plants had 3–4 compound leaves, 5 × 10^7^, 5 × 10^5^, and 5 × 10^3^ CFU/mL of strain ZD01 fermentation broth was sprayed in 20 mL on the eight plants. Untreated plants sprayed with sterile water were used as controls. Then, all the plants were covered with plastic bags. After moisturizing for 24 h, 20 μL of *A. solani* conidia suspension (5 × 10^4^ CFU/mL) was inoculated onto the center of a leaf, and 5–10 leaves were treated per plant. The plastic bags were placed over the plants for moisturizing. At 10 days after plant leaves were inoculated with the *A. solani* conidial suspension, the biocontrol effects of ZD01 were evaluated by measuring diseased leaf rate and lesion area. Diseased leaf rate was calculated in accordance with the following formula:

Diseased leaf rate (%) = (Number of diseased leaves/Total leaves inoculated) × 100%.

### OJIP Transient Measuring Equipment

At 10 days after plant leaves were inoculated with the *A. solani* conidial suspension under greenhouse conditions, leaves treated with different concentrations of strain ZD01 fermentation broth (5 × 10^7^, 5 × 10^5^, and 5 × 10^3^ CFU/mL) were collected respectively for chlorophyll (Chl) α fluorescence emissions detection. Untreated leaves sprayed with sterile water were also collected used as controls. All collected leaves were kept for moisturizing. Chlorophyll (Chl) α fluorescence emissions were measured using a Handy PEA (Handy PEA-Plant Efficiency Analyser, Hansatech Instruments, King’s Lynn, Norfolk, United Kingdom).

Leaves were dark-adapted for 20 min before they were measured. Illumination provided by an array of three light-emitting diodes (LEDs) focused on a diseased leaf area having a diameter of 5 mm for 30 min. Transmission changes at 725–745 nm and Chl α fluorescence were recorded simultaneously using a dual-channel PEA Senior instrument (Hansatech Instruments). Each measurement consisted of three parts. Firstly, initial fluorescence Fo was induced at 0.1 μmol⋅m^–2^⋅s^–1^ for measurement light (pulse frequency of 1 Hz). Then the maximum fluorescence Fm was induced by 4800 μmol⋅m^–2^⋅s^–1^ saturated pulsed light (relaxation time 0.8 s). When the fluorescence intensity dropped from Fm to Fo level, fluorescence kinetic curve was generated. Finally, the chlorophyll fluorescence parameters were calculated.

### *In vivo* Antifungal Activities of the Supernatant, Bacteria and Fermentation Broth of ZD01

For fermentation broth, strain ZD01 was inoculated in 2 mL of LB broth and grown at 37°C with shaking at 200 rpm for 12 h. Then 5 mL of the culture was re-inoculated into 50 mL of fresh LB broth and incubated at 37°C with shaking at 200 rpm for 6 h. The fermentation broth was obtained. For bacteria, the fermentation broth was centrifuged at 8,000 rpm for 30 min at 4°C. The precipitate was collected and resuspended in sterile water. Then the supernatant was also collected and filtered through a 0.22-μm filter membrane (Millex Syringe Filters; Millipore), which was liquid without bacteria.

Healthy potato leaves (‘Helan15’ cultivar) were collected for further experiment *in vivo*. Five to seven pieces of healthy fresh potato leaf were placed onto 1-naphthlcetic acid medium with 0.5% water agar containing 10 μg/mL tetracycline hydrochloride per plate. The potato leaves were treated with fermentation broth, supernatant and bacterial liquid, respectively. The different agents were sprayed onto healthy potato leaves with 5 mL per plate. Leaves treated with sterile water were used as negative controls. Each treatment was performed in six separate dishes. After the leaves were treated, five to seven pieces of healthy fresh potato leaf were placed onto 1-naphthlcetic acid medium with 0.5% water agar containing 10 μg/mL tetracycline hydrochloride per plate. Then, 25 μl of *A. solani* conidia suspension (10^5^ CFU/mL) was inoculated onto the center of each fresh leaf. After 5 days of growth under 12-h light/12-h dark conditions alternately at 25°C, the lesion areas and pathogen copy numbers per leaf were measured. The experiment was repeated three times.

### Effects of *Bacillus subtilis* ZD01 Supernatant, Lipopeptides and Fengycins on Mycelial Growth and Conidial Germination

To assess the inhibitory activity of the supernatant on mycelial growth, a 5-mm diameter plug of *A. solani* HWC-168 was transferred to the center of a PDA plate. A sample of 50 μl supernatant was poured into four holes, 2.5 cm away from *A. solani*, and a blank control of sterile water was included. To test the LP mixture, the LP extract dissolved in methanol (100 μL, 50 mg/mL) was spotted 2.5 cm from the edge of the PDA plate to one side of the fungal plug. Methanol was placed at diametrically opposite points, 2.5 cm from the edge of the PDA plate. For fengycins and surfactins, 50 μL (10 mg/mL) reagents were spotted 2.5 cm from the edge of the PDA plate to one side of the fungal plug. The inhibition zones were measured after 6 days of dual-culturing at 25°C. The experiment was repeated in triplicate.

For conidial germination, 100 μL ZD01 supernatant was mixed with 100 μL spore suspension, and fengycins dissolved in methanol was mixed with 100 μL spore suspension, to 10 μg/mL. The mixtures were spread independently onto 1.0% water agar medium. The spore suspension mixed with sterile water and methanol were used as controls, respectively. The plates were incubated at 25°C for 6–8 h. The experiment was repeated in triplicate. The inhibition of conidial germination was calculated in accordance with the following formula:

Inhibition of conidial germination (%) = (the conidial germination of control − the conidial germination of treatment group)/the conidial germination of control × 100%.

### Quantitative Real-Time PCR Testing of *Alternaria solani* Contents in Potato Leaves

Total RNAs of potato leaves treated with the supernatant, bacteria and fermentation broth of ZD01 were extracted using the Easy Pure Plant RNA Kit (TransGen Biotech, Beijing, China) in accordance with the manufacturer’s instructions. First-strand cDNA was obtained using reverse transcriptase (TransGen Biotech, Beijing, China) using random hexamer primers. Real-time PCR was performed with SYBR Premix Ex Taq (TransGen Biotech). The ITS region was used as an internal reference gene. The relative expressions of specific genes were calculated using the 2^–Δ^ ^Δ^ *^CT^* method (Livak and Schmittgen, 2001).

### Isolation and Purification of Lipopeptides and a Matrix-Assisted Laser Desorption/Ionization Time of Flight Mass Spectrometry Analysis

The LPs were isolated as follows: *B. subtilis* ZD01 was cultured in liquid medium at 37°C for 24 h at 220 rpm, and then, 100 μl of the fermentation culture was transferred into 100 mL LB liquid medium at 37°C for 48 h with shaking. The fermentation broth was centrifuged at 8,000 rpm for 30 min at 4°C, and the supernatant was filtered through a 0.22-μm filter membrane (Millex Syringe Filters; Millipore). The pH of the collected supernatant was adjusted to 2 and stored at 4°C overnight. The crude extract was obtained from the culture broth by centrifugation (8,000 rpm for 30 min at 4°C), and the precipitate was dissolved in methanol. After another centrifugation, the supernatant was collected and vacuum dried, and the residue was collected. The collected residues were then dissolved in methanol. Methanol extracts were vacuum dried using a SCANVAC vacuum concentrator (LaboGene ApS, Lynge, Denmark), and the solid residues were resuspended in a 30% methanol aqueous solution. After the resuspension of the solid residues, the suspensions were filtered through a Cleanert S C18 tube (500 mg/3 mL, 50/pkg) by 90% methanol aqueous solution elution, and the filtrate containing the LP mixture was collected.

The cell-free extracts of LPs obtained from the methanol extractions were subjected to separation using high-performance liquid chromatography. The extract was syringe-filtered using a 0.22 μm nylon filter (Millex Syringe Filters; Millipore). Filtrates were injected into an analytical high-performance liquid chromatograph with a C18 column (5 μm; 250 × 4.6 mm; VYDAC 218 TP) at 30°C connected to an Agilent 6410 Series (Agilent, Palo Alto, CA, United States). The extracted and purified products were analyzed by matrix-assisted laser desorption/ionization time of flight mass spectrometry (MALDI-TOF-MS) using a Bruker Daltonik Reflex MALDI-TOF instrument with a 337-nm nitrogen laser for desorption and ionization (Vater et al., 2003). α-Cyano-4-hydroxycinnamic acid served as the matrix.

### Antifungal Susceptibility Testing for Fengycins

The MIC of the fengycins was detected for *A. solani* using a microdilution method, with some modifications (Wei et al., 2017; Far et al., 2018). The PDB (100 μl/well) was dispensed in each well of a 96-well plate and mixed with fengycins (100 μl/well) and continuously diluted two times with methanol, resulting in final concentrations of fengycins from 0.49 to 250 μg/mL. Hyphae cells were added to each well (3.0 mg/well) and adjusted to 15 mg/mL. In the test, 200 μL of the PDB served as a blank control, and hyphal cells in 200 μl of PDB solution served as a growth control group (Figure 4E). The differences the two was that the former contained only PDB, but not fengycins or hyphal cells; whereas the latter contained both PDB and hyphal cells, but not fengycins. After incubation for 12 h at 25°C, 0.07 g/L resazurin was added to all the wells and incubated for another 4 h to generate clear-cut endpoints. Resazurin was a non-toxic redox dye that has been used extensively to test bacteria and fungi in chemical sensitivity assays (Wei et al., 2017; Far et al., 2018). By visual inspection, a change in color from blue (oxidized state) to pink (reduced state) indicated the growth of fungus, and the MIC was defined as the minimum inhibitory concentration of the compound that prevented this color change.

### Scanning Electron Microscope Observations of Hyphal Morphology

The mycelial morphology of *A. solani* in the control or groups treated with strain ZD01 and fengycins at the MIC value were visualized using SEM. Mycelia of each group were harvested and fixed in 2% glutaraldehyde at 4°C and then dehydrated with gradient ethanol solutions (30%, 50%, 80%, 90%, and 100%). Afterward, ethanol was replaced with 100% tertiary butyl ethanol. Cells were then freeze-dried, coated with gold and imaged using a Hitachi S-3500N field emission SEM (Hitachi, Tokyo, Japan). The experiment was repeated three times.

### Transmission Electron Microscopy

Transmission electron microscopy (TEM) was used to observe internal morphological changes in *A. solani* colonies. The *A. solani* mycelia and conidia were treated with an MIC dose of fengycins, and plates without fengycins were used as controls. Then, hyphae and conidia were collected. The conidia were collected by centrifugation (5,000 × *g*, 15 min, 20°C). Hyphae and conidia were washed and fixed with 2% glutaraldehyde for 30 min at 4°C. The specimens were prepared in accordance to the reference (Yamanaka et al., 2005) for TEM analysis. Ultra-structural changes in the cells were observed using a Hitachi H-7650 transmission electron microscope (Hitachi).

### RNA Extraction and RNA-Seq

The mycelia used in this experiment were collected from fungi cultured on PDA medium for 7 days. The mycelia were added to PDB medium and exposed to the MIC dose of fengycins for 2 and 6 h. The medium without fengycins was used as a control. Each group had three biological replicates. Total RNA was extracted using TRIzol reagent (Invitrogen) in accordance with the manufacturer’s instructions. The quality and integrity of the RNA were determined using a micro-UV Thermo NanoDrop One 2000 spectrophotometer (Thermo Scientific, Waltham, MA, United States) and agarose gel electrophoresis, respectively. Approximately 1 μg of total RNA extracted from mycelia (0, 2 and 6 h) was applied for RNA-Seq. The RNA-Seq was performed using an Illumina HiSeq 4000 platform (Illumina, San Diego, CA, United States) at Novogene Bioinformatics Technology Co., China.

### Transcriptomic Data Analysis

Low quality and joints reads were removed to obtain high-quality clean reads, and these were then mapped to the *Ceratocystis fimbriata* genome using HISAT2 tools software (Kim et al., 2015). The gene expression levels were normalized using fragments per kilobase of transcript per million fragments mapped (Pan et al., 2020). During the identification of differentially expressed genes (DEGs), fold change ≥ 2 and FDR < 0.01 were used as the screening criteria on the basis of DEseq (Wang et al., 2010). To assign the biological processes to DEGs, we performed a GO enrichment analysis of the DEGs in accordance with the GOseq R package, and GO terms with a corrected *P*-value (padj) < 0.05 were considered significantly enriched (Young et al., 2010). To further clarify the advanced functions and connections among the biological system, we carried out a statistical enrichment of DEGs in the KEGG pathways using KOBAS software (Xie et al., 2011). A two-tailed Fisher’s exact test based on the FDR cutoff of 0.05 was used as a justification condition.

### Fluorescence Microscopy Imaging

Green fluorescent signaling in *A*. *solani* was visualized using a Nikon Ti2-U fluorescence microscope (Nikon Corporation, Tokyo, Japan). The samples were immersed in an MIC value dose of fengycins at 25°C for 6 h. The mycelia were collected, and 0.8 μM Sytox Green solution was added. The samples were incubated for 15 min in the dark. Then, mycelia were rinsed two times with 8.5% sterile saline. Samples were examined using a fluorescence microscope. The excitation wavelength was 488 nm, and the emission wavelength was 538 nm (Muoz et al., 2013).

### Determination of the Chitin Contents

The chitin contents of *A. solani* cells in an MIC value dose of fengycins treatments in PDB for 12, 24, 36, and 48 h were determined using the following steps: The samples were dried at 80°C for 3 h and ground into powders. Then, the powders (W1) were treated with a saturated KOH solution at 140°C for 60 min. Afterward, the products were rinsed slowly with distilled water, and centrifuged supernatants were obtained. The extracts were successively dehydrated in 95% and 100% alcohol, and then, they were weighed again (W2). The chitin contents were calculated using the following formula (OuYang et al., 2019):

Chitin content (%) = W2/W1 × 1.26 × 100%,

where 1.26 is the conversion factor.

### Extracellular ATP Measurement Assay

The same design as described above was used to investigate the effects of fengycins on the mycelial ATP contents (Wang et al., 2020). For A. *solani*, 5-mm^2^ plugs of mycelial agar were immersed in an MIC value dose of fengycins for 20, 40, 60, 80, 100, and 120 min at 25°C. Plates without fengycins were used as controls. The *A*. *solani* cells and the supernatants were collected by centrifugation (12,000 × *g*, 5 min, 4°C) independently. The extracellular ATP level was determined using an Enhanced ATP Assay Kit (Beyotime Biotechnology Inc., Shanghai, China) and a multi-function microplate reader (Tecan Spark, Salzburg, Austria). The ATP kit was based on a luminescent ATP assay protocol that involves the lysis of the cell samples, addition of the luciferase enzyme and luciferin, and measurement of the emitted light. The experiment was repeated in triplicate.

### Statistical Analyses

Three independent experiments were performed for each assay. Data were analyzed using SPSS20.0 Windows Software (SPSS Inc., Chicago, IL, United States). Least significant differences were calculated to compare the results at the 0.05 level.

## Results

### Effects of *Bacillus subtilis* ZD01 on the Control of Potato Early Blight Under *in vivo* Conditions

*Bacillus subtilis* ZD01 was selected from 103 isolated *Bacillus* strains that showed the strongest antagonistic activities against *A. solani* in our previous study (Zhang et al., 2020). Then, strain ZD01 was collected to test its potential as a biocontrol agent *in vivo*. To identify the control efficiency of strain ZD01 on *A. solani*, we evaluated the diseased leaf rates and lesion areas of potato plants receiving different treatments under greenhouse conditions.

**FIGURE 1:**
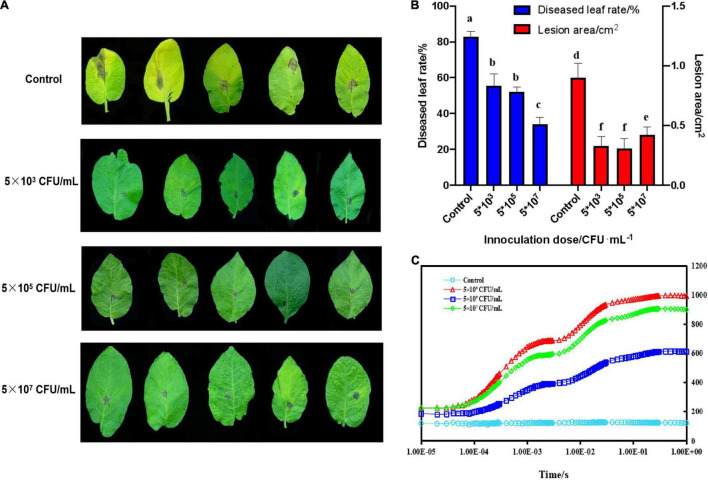
Biocontrol effects of *B. subtilis* ZD01 fermentation broth on the potato early blight in a pot assay under greenhouse conditions. **(A)** Effects of 5 × 10^3^, 5 × 10^5,^ and 5 × 10^7^ CFU/mL of *B. subtilis* ZD01 fermentation broth on the development of early blight symptoms of potato leaves. **(B)** The diseased leaf rate and lesion areas of potato leaves inoculated with *A. solani* HWC-168 with or without the ZD01 fermentation broth treatment. **(C)** Chlorophyll α fluorescence induction of a diseased potato leaf (kept in darkness for 20 min before the measurement). Data are presented as means of three replicates ± SDs, and error bars represent the SDs for three replicates. Means with different letters have significant differences (*p* < 0.05).

As shown in Figure 1A, in control groups, *A. solani* caused leaf chlorosis compared with treated groups. Moreover, the diseased leaf rates of potato plants significantly (*p* < 0.05) decreased when treated with 5 × 10^7^CFU/mL ZD01 fermentation broth to 34.17%, compared with the control group (83.02%). No significant changes were observed with 5 × 10^3^ and 5 × 10^5^CFU/mL treatments. With the control treatment, the lesion areas extended to 0.90 cm^2^, whereas for leaves treated with 5 × 10^3^, 5 × 10^5^ and 5 × 10^7^CFU/mL, the lesion areas were limited to 0.33 cm^2^, 0.31 cm^2^ and 0.42 cm^2^, respectively (Figure 1B). The Chl a fluorescence transient (OJIP) curve indicated that *A. solani* lowered the rate of leaf photosynthesis in its compatible host. The original fluorescence induction kinetics were not observed in potato leaves infected with the fungus in the control groups, which indicated that in potato-infected leave areas no Photosystem II processes occurred (Figure 1C). However, diseased leaves treated with strain ZD01 still had photosynthetic abilities, to some extent, which correlated with the visual severity of the infection (Figure 1A).

### Effects of Secondary Metabolites Produced by *Bacillus subtilis* ZD01 on the Control of Potato Early Blight *in vivo*

**FIGURE 2:**
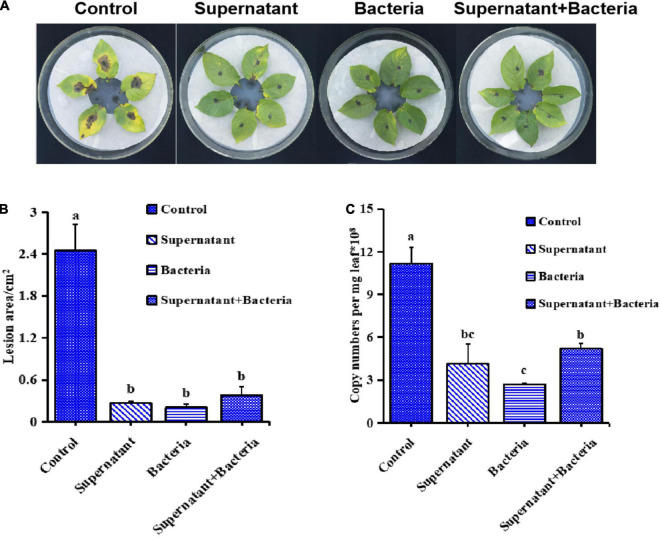
*Bacillus subtilis* ZD01 significantly reduced the disease severity caused by *A. solani* HWC-168 on potato leaves *in vivo*. **(A)** Evidence of disease development on potato leaves treated with the ZD01supernatant, bacteria and fermentation broth prior to *A. solani* HWC-168 inoculation. **(B)** The lesion areas of potato leaves of potato inoculated with *A. solani* HWC-168 and co-cultured with ZD01 supernatant, bacteria and fermentation broth. **(C)** Quantitative detection by qPCR of *A. solani* HWC-168 growth on potato leaves inoculated with *A. solani* HWC-168 and co-cultured with the ZD01 supernatant, bacteria and fermentation broth. Data are presented as means of three replicates ± SDs, and error bars represent the SDs for three replicates. Means with different letters are significantly different (*p* < 0.05).

To further investigate the potential biocontrol functions of *B. subtilis* ZD01, the supernatant, bacteria and fermentation broth were collected to test *in vivo* in leaves. As shown in Figure 2A, the development and expansion of disease symptoms induced by *A. solani* was inhibited effectively by the supernatant, bacteria and fermentation broth of *B. subtilis* ZD01. For leaves treated with the supernatant, bacteria and fermentation broth of ZD01, the lesion areas were limited to 0.27 cm^2^, 0.20 cm^2^ and 0.38 cm^2^, respectively, whereas for leaves in the control group, the lesion areas extended to 2.45 cm^2^ after a 5-day incubation at 25°C (Figure 2B). Then, RT-PCR was used to quantify the content of *A. solani* HWC-168 in potato leaves. The relative pathogen copy numbers per milligram leaf were 4.14 × 10^8^, 2.72 × 10^8^ and 5.21 × 10^8^ for supernatant, bacteria and fermentation broth, respectively (Figure 2C), whereas it was 11.15 × 10^8^ for the control, which corresponded with the lesion area results.

### *Bacillus subtilis* ZD01 Changed the *Alternaria solani* Mycelial Morphology and Inhibited Conidial Germination

**FIGURE 3:**
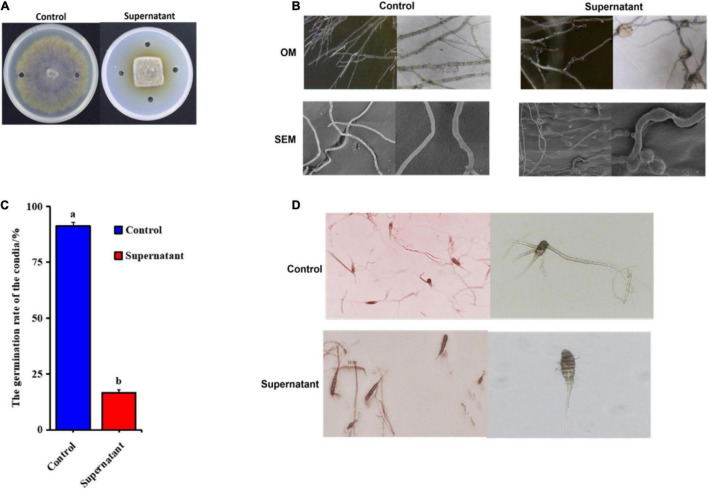
Supernatant extracted from the ZD01 fermentation broth exhibited inhibitory effects on *A. solani* HWC-168 mycelial growth and conidial germination. **(A)** Effects of the supernatant produced by *B. subtilis* ZD01 on *A. solani* mycelial growth. **(B)** Optical and scanning electron micrographs of *A. solani* co-cultured with the ZD01 supernatant. **(C,D)** Reduction in conidial germination of *A. solani* treated with the ZD01 supernatant. Data are presented as means of three replicates ± SDs, and error bars represent the SDs for three replicates. Means with different letters are significantly different (*p* < 0.05).

Because the pathogenicity of *A. solani* decreased greatly after exposure to the ZD01 supernatant, we analyzed the effects of *B. subtilis* ZD01 on *A. solani* hyphae and conidia. Under *in vitro* conditions, *A. solani* was cultured for 5 days with *B. subtilis* ZD01 supernatant on potato dextrose agar (PDA) and assessed using a dual-culture assay. Compared with the control, the *B. subtilis* ZD01 supernatant significantly reduced the growth of *A. solani* (Figure 3A). In addition, the supernatant released by *B. subtilis* ZD01 showed a broad inhibitory range against plant pathogens (Supplementary Figure 1).

**FIGURE 4:**
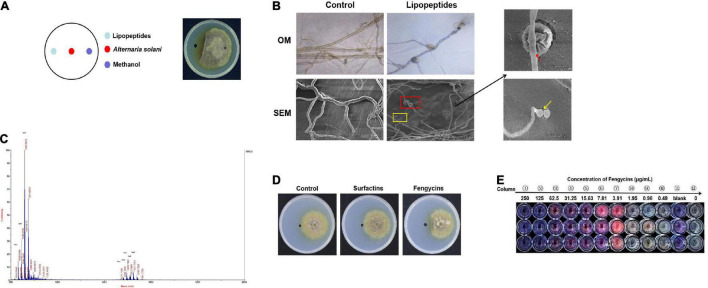
Lipopeptides produced by ZD01 show antagonistic effects against the growth of *A. solani* HWC-168. **(A)** Colony morphology of *A. solani* co-cultured with ZD01 lipopeptides. **(B)** Optical and scanning electron micrographs of *A. solani* co-cultured with ZD01 lipopeptides. **(C)** Identification of ZD01 lipopeptides by MALDI-TOF-MS. **(D)** Colony morphology of *A. solani* co-cultured with fengycins and surfactins for the analysis of the active ingredients against *A. solani*. **(E)** Determination of the MIC value of fengycins against *A. solani* using the microdilution method. The red arrows indicate wrinkled surfaces hyphal cells treated with lipopeptides; the yellow arrows indicate the vacuolation of hyphae after exposure to lipopeptides.

Mycelial structures play vital roles in the infection process. Thus, to elucidate whether biocontrol agent *B. subtilis* ZD01’s ability to inhibit *A. solani* mycelial growth is related to the hyphal malformation, optical microscopy (OM) and scanning electron microscopy (SEM) were used to analyze the detailed morphological changes in *A. solani* hyphae. Using OM, numbers of obviously enlarged mycelia were observed in the treated groups (Figure 3B). Using SEM, it was observed that, for the control group, the normal hyphae exhibited regular lengths, smooth surfaces and intact structures (Figure 3B). However, the mycelia of *A. solani* treated with the ZD01 supernatant showed wrinkled surfaces and deformities (Figure 3B). Abnormal swelling and branches developed along the hyphae after exposure to the supernatant of ZD01 (Figure 3B). The formation of protoplast balls of hyphae also appeared in the treated group (Figure 3B). Therefore, the morphological changes in hyphae may result in the invasive capabilities of hyphae and lessen the mycelial extension into host tissues, leading to reduced pathogenicity.

Spores are also a crucial factor in the infection processes of fungal pathogens; therefore, suppression activity of *B. subtilis* ZD01 on conidial germination was evaluated. As shown in Figure 3C, *B. subtilis* ZD01 significantly inhibited the conidial germination of *A. solani* HWC-168 (*p* < 0.05), whereas the conidial germination rates were 91.33% and 16.67% for the control and treated groups, respectively. In the control group, conidia germinated and formed regular germ tubes (Figure 3D). However, some conidia co-cultured with the ZD01 supernatant formed much shorter germ tubes, and some conidia could not germinate completely, resulting in bubble structures on the sides of *A. solani* conidia (Figure 3D). If conidia could not germinate or form normal germ tubes, then they are not able to produce appressoria and form infection structures, such as penetration pegs, which decreases the probability of a successful infection of *A. solani*. Therefore, these results explained the action mechanisms used by *B. subtilis* ZD01 to reduce *A. solani* virulence.

### Fengycins Are the Major Active Antifungal Metabolites Secreted by *Bacillus subtilis* ZD01

The *B. subtilis* ZD01 supernatant showed strong antifungal effects on *A. solani* mycelial morphology and conidial germination. Consequently, we wanted to elucidate the main active substances in the ZD01 supernatant. Because LPs are the main antifungal secondary metabolites of *Bacillus* species, we analyze the antifungal activities of LPs produced by strain ZD01. These LPs significantly inhibited the mycelial growth of *A. solani* (Figure 4A). To further reveal the antifungal activities of these LPs, the *A. solani* hyphal morphology after being treated with LPs was observed using OM and SEM. As shown in Figure 4B, mycelia in the treated group showed protoplast shrinkage. Most of the *A. solani* cells showed characteristic vacuolation (Figure 4B). These abnormal structures that appeared in the treated group might result in the leakage of cytoplasmic components, which indicated that LPs produced by ZD01 had strong antagonistic effects on fungal pathogens, consistent with the effects of the ZD01 supernatant.

Matrix-assisted laser desorption/ionization time of flight mass spectrometry was used to identify the active compounds in the ZD01 LPs. Table 1 provided an example on how we identified the LPs (Luo et al., 2015). As shown in Figure 4C, m/z 1030.59 [M + Na^+^] was unequivocally identified as C_13_ surfactin A. Molecular ion peak [M + Na^+^] at m/z 1044.61 and 1058.62 corresponded to C_14_ surfactin B and C_15_ surfactin C, respectively. Likewise, molecular ion peaks [M + Na^+^] at m/z 1471.73, 1485.75, 1499.74, 1512.77, and 1527.78 were corresponding to fengycins (fengycin A-fengycin E). This pattern of LPs corresponds to the metabolite spectra found for most surfactin- and fengycin-producing *Bacillus* strains. Therefore, *B. subtilis* ZD01 produces C_13_ to C_15_ surfactins and C_15_ to C_17_ fengycins, identified as the major ingredients of LPs emitted from ZD01.

**TABLE 1:** Calculated mass values of M, M + H^+^, and M + Na^+^ ions corresponding to identified isoforms of surfactins and fengycins in culture extracts from *B. subtilis* ZD01.

Lipopeptide	Mass value*^a^*		

	M	M + H^+^	M + Na^+^
Surfactin A (C_13_)	1,007.6	1,008.6	1,030.6
Surfactin B (C_14_)	1,021.7	1,022.7	1,044.7
Surfactin C (C_15_)	1,035.7	1,036.7	1,058.7
Fengycin A (C15/Ala-6)	1,448.8	1,449.8	1,471.9
Fengycin B (C_16_/Ala-6)	1,462.9	1,463.9	1,485.9
Fengycin C (C_17_/Ala-6)	1,476.9	1,477.9	1,499.9
Fengycin D (C_16_/Val-6)	1,490.9	1,491.9	1,513.9
Fengycin E (C_17_/Val-6)	1,504.9	1,505.9	1,527.9

*^a^The data were compiled from whole cells grown on LB agar.*

Then, we analyzed the antagonistic activities of these LPs toward *A. solani* growth. The fengycins were more potent than the surfactins against *A. solani* growth, producing inhibition zones of 1.07 cm and 0.37 cm, respectively (Figure 4D). Therefore, our results indicated that fengycins were the major active ingredients that exhibited the strongest inhibitory activities among the compounds produced by ZD01.

Mycelia were inoculated into a multiwell plates containing potato dextrose broth (PDB) medium supplemented with the demonstrated concentrations of fengycins, and resazurin was used to generate clear-cut endpoints. As shown in Figure 4E, the mycelial growth of *A. solani* was almost suppressed at 7.81 μg/mL fengycins compared with the 0 μg/mL fengycins group and the growth control group. Fengycins had no effects on the growth of *A. solani* at concentrations lower than 1.95 μg/mL. Therefore, the MIC of fengycins on *A. solani* was 3.91 μg/mL, and we chose the MIC value to explore the antagonistic mechanisms of fengycins against *A. solani*.

### Identification and Functional Annotation of Differentially Expressed Genes After *Alternaria solani* Exposure to Fengycins Treatments

To study the molecular mechanisms of fengycins produced by *B. subtilis* ZD01 involved in the inhibition of *A. solani*, a transcriptome analysis in response to biotic stress was performed. *Alternaria solani* (CK_2h, CK_6h) and *A. solani* vs. fengycins (FenT_2h, FenT_6h) transcriptomes were created in triplicate. The profiles of transcriptome sequence data are shown in Table 2. Using RNA-Seq, an average of 23.47, 22.63, 23.24, and 23.15 million raw reads were generated from CK_2h, CK_6h, FenT_2h and FenT_6h samples, respectively. After filtering the adaptor sequences, the average clean reads were 23.00, 22.13, 22.75, and 22.61 million for the four treatment groups, respectively. In conclusion, the results indicated that the RNA-Seq was of a good quality and was applicable for further analyses.

**TABLE 2:** Profile of the transcriptome sequence data.

Parameter	CK_2h	CK_6h	FenT_2h	FenT_6h
Raw reads (million)	23.47 ± 0.68	22.63 ± 0.88	23.24 ± 0.62	23.15 ± 0.75
Clean reads (million)	23.00 ± 0.57	22.13 ± 0.84	22.75 ± 0.53	22.61 ± 0.82
Clean bases (G)	6.90 ± 0.17	6.64 ± 0.25	6.83 ± 0.16	6.79 ± 0.25
Error (%)	0.03 ± 0.01	0.03 ± 0.01	0.03 ± 0.00	0.03 ± 0.00
Q20 (%)	98.17 ± 0.48	97.96 ± 0.16	97.89 ± 0.08	97.96 ± 0.06
Q30(%)	94.56 ± 1.34	93.98 ± 0.30	93.86 ± 0.17	93.97 ± 0.11
GC content (%)	55.11 ± 0.10	55.01 ± 0.04	55.17 ± 0.03	54.81 ± 0.14
Total mapped reads	42550511(92.51 ± 0.12%)	41277661(93.25 ± 0.38%)	41906706(92.10 ± 0.28%)	42061125(92.99 ± 0.33%)

**FIGURE 5:**
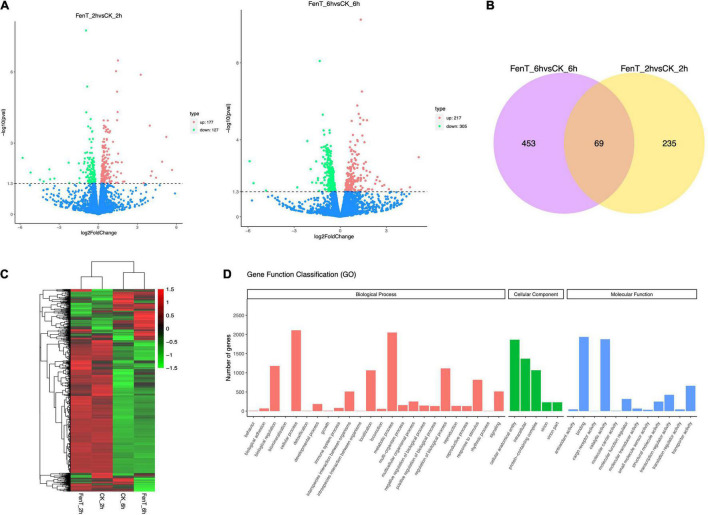
Volcano plots, Venn diagrams, a heatmap and the GO functional classification. **(A)** The differences in the distribution and density distribution of gene expression in CK_2h, CK_6h, FenT_2h, and FenT_6h. **(B)** Venn diagrams showing the numbers of common DEGs that are shared in comparisons among CK_2h, CK_6h, FenT_2h, and FenT_6h. **(C)** Heatmap of the common DEGs in the three treatment groups. **(D)** GO functional classification results of DEGs shared by the three treatment groups. The DEGs were assigned to three categories: cellular component, molecular function and biological process.

The differences in the distribution and density distribution of gene expression in CK_2h, CK_6h, FenT_2h, and FenT_6h samples are shown in Figure 5A. Most of the genes were expressed at low levels, but a few genes were expressed at high levels, and they showed the same gene expression characteristics among the four groups (Figure 5A). Under the effect of fengycins produced by *B. subtilis* ZD01, the expression patterns of *A. solani* genes changed significantly. To learn more about the DEGs between control and fengycin-treated samples, the differential expression of transcripts are shown using a Venn diagram in Figure 5B. Comparisons of gene expression between control and fengycin-treated samples revealed that 304 and 522 genes were significantly differentially expressed at 2 and 6 h, respectively [false discovery rate (FDR) < 0.05] (Figure 5B). Under the stress of fengycins exposure for 2 h, 177 DEGs were up-regulated, whereas 127 DEGs were down-regulated. The inhibition by fengycins for 6 h also caused the significant differential expression of genes, with 217 and 305 genes being up-regulated and down-regulated, respectively. There were 688 genes differentially expressed at 2 and 6 h, and their profiles are illustrated in Figure 5C. Pairwise comparisons showed that the largest number of genes had changed expression levels at 6 h, and this included a statistically significant reduction in expression. In summary, fengycins induced resistance against *A. solani* by regulating the transcription levels of some related genes.

The proportions of up-regulated and down-regulated DEGs shared by the three treatment groups were divided into the three main functional categories (Figure 5D) of the gene ontology (GO) analysis. The top three enriched terms within the biological process category were cellular process, metabolic process and biological regulation. Within cellular components, many genes were involved in cellular anatomical entity, intracellular and protein-containing complex. Within molecular function, many genes were involved in binding and catalytic activity, which included the categories of cell components. To search for genes associated with the processed responses, the DEGs were mapped to terms in the Kyoto Encyclopedia of Genes and Genomes (KEGG) database.

### Pathway Enrichment Analysis for Differentially Expressed Genes Identified in *Alternaria solani* After Exposure to Fengycins

In the KEGG analysis, the DEGs in samples treated with fengycins for 2 h and 6 h were mapped to 53 and 57 KEGG pathways, respectively (Table 3). The two groups had the same KEGG patterns, mainly in cell wall, cell membrane, genetic information, amino acid metabolic and energy metabolic pathways. In detail, cell wall metabolic pathways were mostly amino sugar and nucleotide sugar metabolism and galactose metabolism. The pathways that were involved in cell membrane were sphingolipid, glycerophospholipid metabolism, inositol phosphate metabolism, ether lipid metabolism, fatty acid degradation and steroid biosynthesis. The pathways related to genetic information were ribosome, ribosome biogenesis in eukaryotes, spliceosome, DNA replication, protein processing in endoplasmic reticulum, nucleotide excision repair, RNA transport, aminoacyl-tRNA biosynthesis and mRNA surveillance.; The energy metabolic pathways were mostly nitrogen metabolism, starch and sucrose metabolism, glycolysis/gluconeogenesis, pyruvate metabolism, pentose phosphate pathway, peroxisome and ubiquitin mediated proteolysis, and the pathways related to amino acid metabolism were tryptophan metabolism, tryptophan metabolism, tryptophan metabolism, beta-alanine metabolism and tyrosine metabolism. In addition, there were differences between the expression patterns at 2 h and 6 h. The genes involved in RNA degradation, valine, leucine and isoleucine degradation, propanoate metabolism, protein export, ascorbate and aldarate metabolism, cysteine and methionine metabolism and glycerolipid metabolism that were connected to genetic information and amino acid metabolic processing were specifically expressed at 2 h. The genes involved in proteasome, fructose and mannose metabolism, MAPK signaling pathway-yeast, sulfur metabolism, selenocompound metabolism, alanine, aspartate and glutamate metabolism, cyanoamino acid metabolism, ABC transporters, phenylalanine, tyrosine and tryptophan biosynthesis, homologous recombination, regulation of mitophagy - yeast and glycine, and serine and threonine metabolism that were involved in cell membrane, genetic information and amino acid metabolic pathways were only differentially expressed at 6 h.

**TABLE 3:** Mostly enriched KEGG pathway of DEGs in *A. solani*.

2 h				6 h			

Pathway	Input number	Background number	Pathway ID	Pathway	Input number	Background number	Pathway ID
Fatty acid degradation	4	10	ko00071	Proteasome	4	8	ko03050
Tryptophan metabolism	5	18	ko00380	mRNA surveillance pathway	6	17	ko03015
RNA degradation	4	14	ko03018	Steroid biosynthesis	4	12	ko00100
Valine, leucine and isoleucine degradation	3	10	ko00280	Aminoacyl-tRNA biosynthesis	4	13	ko00970
DNA replication	3	11	ko03030	Starch and sucrose metabolism	8	33	ko00500
Propanoate metabolism	2	7	ko00640	Amino sugar and nucleotide sugar metabolism	6	24	ko00520
beta-Alanine metabolism	3	15	ko00410	Spliceosome	7	30	ko03040
Pyruvate metabolism	2	10	ko00620	Galactose metabolism	4	15	ko00052
Glycolysis/Gluconeogenesis	3	19	ko00010	Nitrogen metabolism	2	6	ko00910
RNA polymerase	2	11	ko03020	Methane metabolism	2	6	ko00680
Basal transcription factors	2	11	ko03022	Glutathione metabolism	3	11	ko00480
Purine metabolism	4	29	ko00230	Basal transcription factors	3	11	ko03022
Steroid biosynthesis	2	12	ko00100	Meiosis - yeast	5	22	ko04113
Pyrimidine metabolism	3	22	ko00240	Ubiquitin mediated proteolysis	5	24	ko04120
Arginine and proline metabolism	2	14	ko00330	Glycolysis/Gluconeogenesis	4	19	ko00010
Protein export	1	5	ko03060	Ether lipid metabolism	2	8	ko00565
Ascorbate and aldarate metabolism	1	5	ko00053	Endocytosis	6	31	ko04144
Cysteine and methionine metabolism	1	5	ko00270	Arginine and proline metabolism	3	14	ko00330
Amino sugar and nucleotide sugar metabolism	3	24	ko00520	Glycerophospholipid metabolism	4	20	ko00564
Phenylalanine metabolism	2	15	ko00360	Pentose phosphate pathway	2	9	ko00030
Nitrogen metabolism	1	6	ko00910	Fructose and mannose metabolism	2	9	ko00051
Methane metabolism	1	6	ko00680	Histidine metabolism	2	9	ko00340
Lysine degradation	2	16	ko00310	Phenylalanine metabolism	3	15	ko00360
Nucleotide excision repair	2	17	ko03420	Cell cycle - yeast	5	28	ko04111
Arginine biosynthesis	1	8	ko00220	Fatty acid degradation	2	10	ko00071
Ether lipid metabolism	1	8	ko00565	Mismatch repair	2	10	ko03430
Pentose and glucuronate interconversions	2	19	ko00040	Purine metabolism	5	29	ko00230
Endocytosis	3	31	ko04144	MAPK signaling pathway - yeast	5	30	ko04011
Pentose phosphate pathway	1	9	ko00030	Sphingolipid metabolism	2	11	ko00600
Butanoate metabolism	1	9	ko00650	RNA polymerase	2	11	ko03020
Glycerolipid metabolism	1	9	ko00561	Tyrosine metabolism	3	18	ko00350
Histidine metabolism	1	9	ko00340	Tryptophan metabolism	3	18	ko00380
Mismatch repair	1	10	ko03430	Sulfur metabolism	1	5	ko00920
RNA transport	3	34	ko03013	Selenocompound metabolism	1	5	ko00450
Oxidative phosphorylation	2	23	ko00190	Alanine, aspartate and glutamate metabolism	2	13	ko00250
Protein processing in endoplasmic reticulum	3	35	ko04141	Cyanoamino acid metabolism	1	6	ko00460
Sphingolipid metabolism	1	11	ko00600	ABC transporters	1	6	ko02010
Glutathione metabolism	1	11	ko00480	Phenylalanine, tyrosine and tryptophan biosynthesis	1	6	ko00400
Ribosome biogenesis in eukaryotes	2	26	ko03008	RNA transport	5	34	ko03013
Aminoacyl-tRNA biosynthesis	1	13	ko00970	Ribosome	2	14	ko03010
Ribosome	1	14	ko03010	Protein processing in endoplasmic reticulum	5	35	ko04141
Cell cycle - yeast	2	28	ko04111	Homologous recombination	1	7	ko03440
Galactose metabolism	1	15	ko00052	Phagosome	2	15	ko04145
Phagosome	1	15	ko04145	Regulation of mitophagy - yeast	2	15	ko04139
Inositol phosphate metabolism	1	15	ko00562	Oxidative phosphorylation	3	23	ko00190
Peroxisome	1	16	ko04146	Arginine biosynthesis	1	8	ko00220
mRNA surveillance pathway	1	17	ko03015	Peroxisome	2	16	ko04146
Tyrosine metabolism	1	18	ko00350	Lysine degradation	2	16	ko00310
Starch and sucrose metabolism	2	33	ko00500	Nucleotide excision repair	2	17	ko03420
Glycerophospholipid metabolism	1	20	ko00564	Butanoate metabolism	1	9	ko00650
Meiosis - yeast	1	22	ko04113	Ribosome biogenesis in eukaryotes	3	26	ko03008
Ubiquitin mediated proteolysis	1	24	ko04120	Pyruvate metabolism	1	10	ko00620
Spliceosome	1	30	ko03040	Pentose and glucuronate interconversions	2	19	ko00040
				DNA replication	1	11	ko03030
				Pyrimidine metabolism	2	22	ko00240
				beta-Alanine metabolism	1	15	ko00410
				Inositol phosphate metabolism	1	15	ko00562
				Glycine, serine and threonine metabolism	1	17	ko00260

In particular, cell wall and membrane metabolic pathways, such as amino sugar and nucleotide sugar metabolism, steroid biosynthesis, ether lipid metabolism and glycerolipid metabolism, were highly enriched in the KEGG analysis. The antifungal actions of fengycins are associated with their incorporation into biological membranes. Chitin is a critical component of the fungal cell walls and plays a key role in the structural integrity of the cell wall. The KEGG annotation showed that amino sugar and nucleotide sugar metabolic pathways were greatly affected by the fengycin treatments. This finding was consistent with the structural changes in the *A. solani* hyphae, in which the cells exposed to fengycins became swollen. More chitin was needed for the inflated hyphae to synthesize cell walls. Therefore, we speculated that the inhibitory mechanisms of fengycin produced by ZD01 against *A. solani* was related to cell wall and membrane damage.

### Fengycins Changed the Cell Wall Integrity and Cell Membrane Permeability of *Alternaria solani*

**FIGURE 6:**
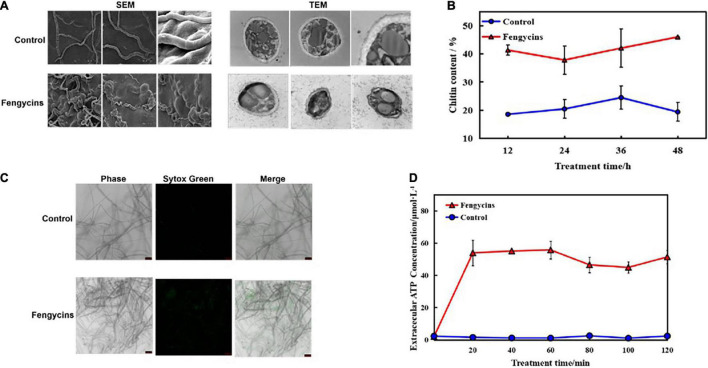
Effects of fengycins on *A. solani* hyphal cell wall integrity and cell membrane permeability. **(A)** Scanning and transmission electron micrographs of *A. solani* hyphae co-cultured with fengycins. **(B)** Chitin contents of *A. solani* hyphae co-cultured with fengycins and a control groups. **(C)** Fluorescence microscope imaging of *A. solani* hyphae treated with fengycins and a control groups. The phase channel shows all the fungal cells in bright-field images, and the Sytox Green channel shows cells attached or inserted with Sytox Green labeling; the merged channel shows the proportion of Sytox Green-labeled cells. **(D)** Effects of fengycins on ATP release from *A. solani*. The changes in the extracellular ATP levels of *A. solani* represent cell membrane damage. The results are presented as the means ± SDs (*n* = 3).

Fungal cell walls are dynamic organelles required to maintain cell shape and protect against environmental damage. They also provide recognition functions for the innate immune system and are potential molecular targets of antifungal therapies (Gozalbo et al., 2004). The cell membrane is double-layered and contains lipids and proteins that give the cell structure and regulate the transport of substances across the cell. To explore whether fengycins functioned on *A. solani* cell walls and membranes, we used SEM and transmission electron microscope (TEM) to observe morphological changes. As shown in Figure 6A, the micro-morphology of *A. solani* was significantly changed by the fengycins treatments. Mycelia of the control group were of a regular length and had intact structures with smooth surfaces. However, the hyphae of the treatment samples were swollen and contained imperfect cells. In addition, the TEM observation revealed that fengycins caused hyphae to become shrunken, the cell walls and membranes became thinner, and some of the cell membranes appeared damaged (Figure 6A). The structural destruction of the hyphae might lead to the leakage of cell contents.

The chitin contents of *A. solani* mycelia rapidly increased (*p* < 0.05) along with fengycins exposure. After 48 h of exposure, the chitin content in the fengycins-treated samples was 46.02%, which was significantly higher than the control samples (19.42%) (*p* < 0.05) (Figure 6B).

The effects of fengycins on the cell membrane integrity of *A. solani* were determined using fluorescent microscopy as shown in Figure 6C. When mycelia were treated with 10% methanol without fengycins, almost every hypha was intact without green fluorescent lines. However, more green fluorescence lines were observed in the fengycin-treated groups than in the control groups when treated at the same time.

Moreover, ATP is produced in the cytoplasm and usually locates in the periplasmic space. If the permeability of a cell wall is impaired, then ATP is released from fungal cells into the intercellular spaces. Therefore, we used the ATP content as an indicator to analyze the effects of fengycins on the cell membranes of fungal hyphae. As shown in Figure 6D, the extracellular ATP contents in the treated samples increased rapidly after 20 min. After 60 min of exposure, the chitin content in the fengycin-treated samples was 55.73 μmo/L, which was significantly higher than in the control samples (1.10 μmol/L). In contrast, the extracellular ATP levels of the control cells were in a constant low range from 20 to 120 min. Thus, the results indicated that fengycins damaged the structures and changed the cell membrane permeability of *A. solani*.

These results revealed that the normal metabolic processes related to the cell wall and membrane were disrupted by fengycins, which finally damaged the cell wall and disrupted the membrane integrity.

### Fengycins Exhibited Strong Effects on *Alternaria solani* Conidia

**FIGURE 7:**
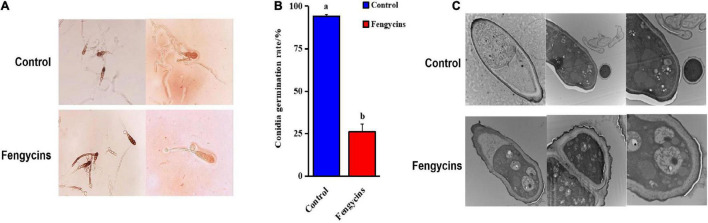
Effects of fengycins on conidial germination and structure. **(A,B)** Reduction in the conidial germination of *A. solani* after treatment with fengycins. **(C)** Transmission electron micrographs of *A. solani* co-cultured with fengycins. Data are presented as means of three replicates ± SDs, and error bars represent the SDs for three replicates. Means with different letters are significantly different (*p* < 0.05).

In addition to mycelia, conidial vitality plays a crucial role during fungal pathogen infections. Here, we analyzed the effects of fengycins on *A. solani* conidia. For the control groups, conidia formed normal-length germ tubes. However, for the treated groups, the fungal conidia could not germinate or formed short germ tubes after exposure to fengycins. Some vacuolation was observed in the treated conidia (Figure 7A). Fengycins strongly inhibited the conidial germination of A. solani, resulting in 94.00% and 26.00%, respectively, for the control and treated groups (Figure 7B).

Because fengycins showed strong antagonistic effects on conidia, we speculated that fengycins might change the conidial integrity. As shown in Figure 7C, all the conidial cells became hollow, and the number of organelles decreased and even disappeared. The conidia treated with fengycins appeared to have thinner cell walls and membranes, and some of the cell walls and membranes were damaged. In particular, the mitochondria in the conidia swelled and the presence of bubbles increased.

## Discussion

Our previous study identified an efficient strain, *B. subtilis* ZD01, that effectively controlled *A. solani*. In this study, the active compounds produced by *B. subtilis* ZD01 could suppress the growth and conidial germination of *A. solani in vitro*, and further reduced the fungal pathogenicity *in vivo*. We found that fengycins were the active ingredients in the ZD01 supernatant that inhibited *A. solani* growth. Therefore, fengycins were used to further investigate the possible antifungal mechanisms against *A. solani* using transcriptome profiles determined by an RNA-Seq analysis. A large number of gene expression levels and metabolic pathways were influenced by the fengycins. The expression patterns of DEGs at 2 h and 6 h were similar, including the metabolic processes related to cell wall, cell membrane, energy, amino acid and genetic information. However, there were some differences among the expression pathways between the 2-h and 6-h treatments that were related to genetic information processing and amino acid metabolism, which indicated that the effects of fengycins on *A. solani* differed depending on the various cellular states. This funding was consistent with the effects of the oil decanal against *Penicillium expansum* treated for different times (Zhou et al., 2018).

*Bacillus* strains produce antagonistic metabolites that target pathogens effectively. As the major type of antibiotics produced by *Bacillus* species, LP biosurfactants play essential roles in inhibiting the growth of plant pathogens, and they include three families, e.g., surfactins, iturins and fengycins (Ongena and Jacques, 2008). In particular, fengycins showed significant antifungal activities against a broad range of fungi, such as *Botrytis cinerea* (Toure et al., 2004), *Fusarium graminearum* (Wang et al., 2007) and *Podosphaera fusca* (Romero et al., 2007). These compounds cause morphological changes in fungal plasma membranes and cell walls (Tendulkar et al., 2007; Zhang et al., 2018). Determining the mode of action is crucial and necessary for developing *Bacillus* strains for the biocontrol of potato early blight disease. Therefore, the mechanism was investigated at the transcriptional level in this study. The fengycins influenced the expression of a large number of genes related to cell wall and membrane, including those of amino sugar and nucleotide sugar metabolism, galactose metabolism and glycerophospholipid metabolism. Moreover, fengycins produced by ZD01 induced structural deformities and changed the cell membrane permeability of the fungal mycelia. Therefore, we hypothesized that fengycins fulfill the essential regulatory functions to target cell walls and membranes.

Cell walls are important in sustaining cell morphology and protecting cells against life-threatening environmental conditions (Bowman and Free, 2006; Ruiz-Herrera et al., 2006). The integrity of cell walls is highly important to many metabolic processes. Chitin, a β-(1,4)-linked polymer of *N*-acetylglucosamine, is an important structural component of the cell walls of filamentous fungi that plays critical roles in fungal development and pathogenicity (Klis, 1994). In our study, the chitin contents of the cell walls were affected by fengycins treatments, which was confirmed by the chitin contents assay. These results demonstrated that the cell wall was an important target of the fengycins.

Cell membranes are also crucial for maintaining cell viability because they form barriers that separate the cells from their surroundings, and they have channels for exchanging substances and energy between the cell and the surrounding environment (Shao et al., 2013; Tao et al., 2014). The permeability and fluidity of the membrane can be affected, which is of considerable significance to the survival of cells (De Lira Mota et al., 2012). Consistent with the previous studies (González-Jaramillo et al., 2017), fengycins impaired the membrane integrity of *A. solani*. After the membrane was destroyed, cell contents easily passed through the cell membrane. In addition to acting on the cell wall, the results suggested that fengycins also worked on the cell membrane.

## Conclusion

**FIGURE 8:**
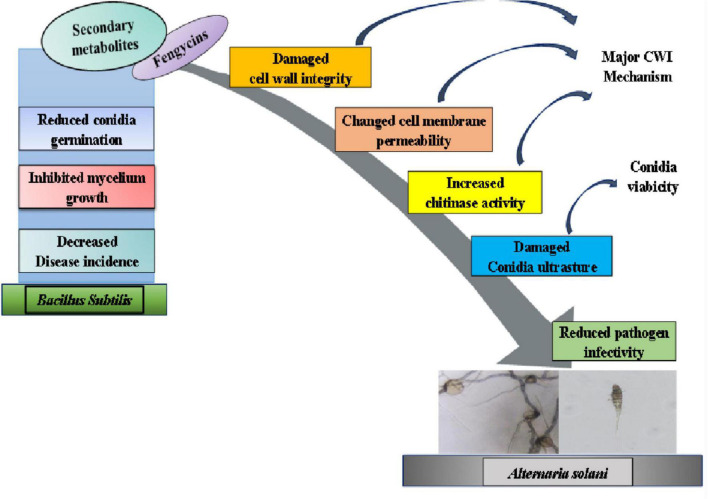
A model for the mode of action of fengycins produced by ZD01 against *A. solani*. Fengycins produced by ZD01 mediate *A. solani* mycelial growth and conidial germination. Fengycins damage the cell wall integrity, change mycelial cell membrane permeability and alter conidial structures, which subsequently leads to the suppression of fungal growth, mycelial penetration, conidial vitality and germination. Therefore, *A. solani* fails to infect potato leaves.

In summary, secondary metabolites secreted by *B. subtilis* ZD01 effectively limited the development of lesion area of potato early blight and caused mycelial and conidial malformations, which was then demonstrated that fengycins were the key active antifungal compounds. The RNA-sequencing data revealed that fengycins affected multiple metabolic processes related to cell walls and membranes, such as steroid biosynthesis, fatty acid metabolism and glycerophospholipid metabolism. Besides, the biochemical analyses in the present study suggested that fengycins damaged cell wall integrity and changed cell membrane, which was consistent with the transcriptome data. Taken together, fengycins’ modes of action against *A. solani* lead to functional degradation by damaging the cell wall integrity, altering cell membrane permeability and damaging the conidial structures (Figure 8). The current work provided important information for the development of fengycins as a potential chemical to control potato early blight disease.

## Data Availability Statement

The datasets presented in this study can be found in online repositories. The names of the repository/repositories and accession number(s) can be found below: https://www.ncbi.nlm.nih.gov/nuccore/CP046448; https://www.ncbi.nlm.nih.gov/assembly/GCA_002837235.1/.

## Author Contributions

DZn, RQ, YP, and SY performed the experiments. DZn and RQ wrote the manuscript. ZZ and YP revised the manuscript critically for important intellectual content. SY, WY, and JC provided data curation and methodology. JW, DZo, and YP provided technical assistance. DZn, JZ, and ZY designed the experiments. JZ and ZY provided supervision and project administration.

## Conflict of Interest

The authors declare that the research was conducted in the absence of any commercial or financial relationships that could be construed as a potential conflict of interest.

## Publisher’s Note

All claims expressed in this article are solely those of the authors and do not necessarily represent those of their affiliated organizations, or those of the publisher, the editors and the reviewers. Any product that may be evaluated in this article, or claim that may be made by its manufacturer, is not guaranteed or endorsed by the publisher.
