# Supplementary material for: Biocontrol and Action Mechanism of Bacillus subtilis Lipopeptides’ Fengycins Against Alternaria solani in Potato as Assessed by a Transcriptome Analysis
Source: Front Microbiol. 2022 May 11;13:861113. doi: 10.3389/fmicb.2022.861113 (PMC9130778; doi:10.3389/fmicb.2022.861113)
Supplement: Supplementary file 1 [file Data_Sheet_1.doc]

**Table S1 Strains used in this study**

| **Strain** | **Description** | **Source** |
| --- | --- | --- |
| *Bacillus subtilis* ZD01 | Undomesticated environmental strain | Lab strain collection |
| *Alternaria solani* HWC-168 | Undomesticated environmental strain | (Zhang, He et al. 2018) |
| *Bipolaris sorokinianum* HA-B | Undomesticated environmental strain | Lab strain collection |
| *Rhizoctonia solani* HA-R | Undomesticated environmental strain | Lab strain collection |
| *Botrytis cinerea* HA-12 | Undomesticated environmental strain | Lab strain collection |
| *Aternaria mali roberts* HA-3 | Undomesticated environmental strain | Lab strain collection |
| *Alternaria alternata* B7 | Undomesticated environmental strain | Lab strain collection |
| *Streptomyces scabies* HP4 | Undomesticated environmental strain | Lab strain collection |


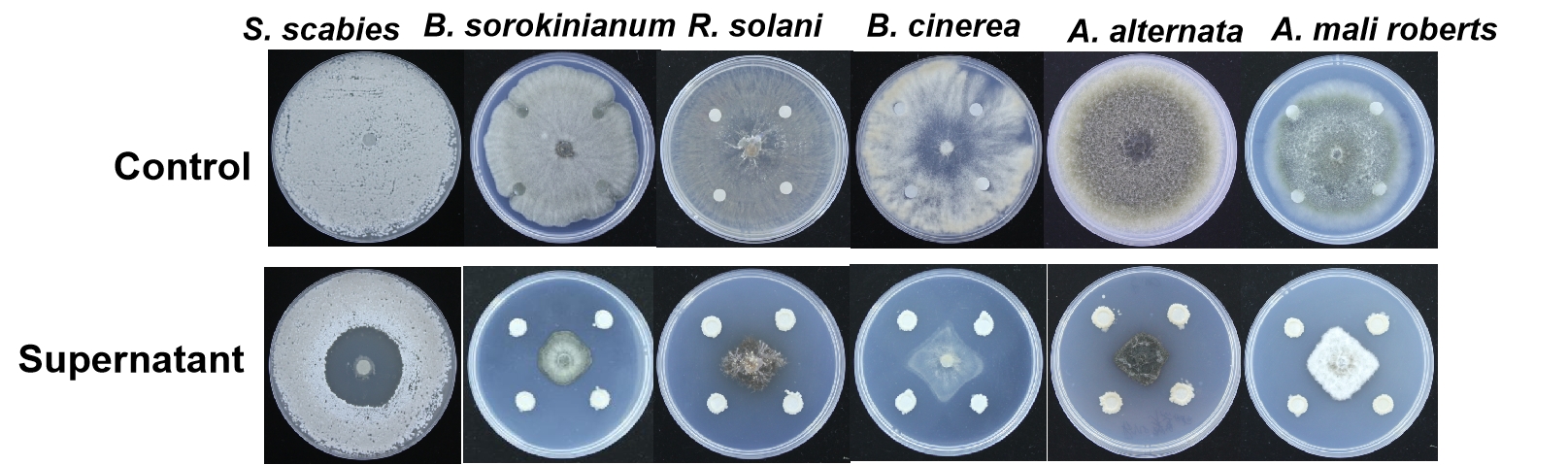


Figure S1. Antagonistic activity of supernatant produced by ZD01 supernatant against six plant pathogens, including *Streptomyces scabies*, *Bipolaris sorokinianum*, *Rhizoctonia solani*, *Botrytis cinerea*, *Alternaria alternata*, and *Alternaria mali roberts*.
